# Cardiothoracic Surgery Insights From a Five-Year Cohort Study in a Low-Volume Referral Center

**DOI:** 10.7759/cureus.105225

**Published:** 2026-03-14

**Authors:** Bosco A Matarrita-Quesada, Sebastián Rojas-Chaves, Klaus Kuhn-Delgadillo, Ann Echeverri-McCandless, Jose M Sprok-Tromp, Irene Bolaños-Aguiar, Natasha Salazar-Durón, Alfredo Sanabria-Castro

**Affiliations:** 1 Cardiothoracic Surgery, Hospital San Juan de Dios-Caja Costarricense de Seguro Social (HSJD-CCSS), San José, CRI; 2 Research, Hospital San Juan de Dios-Caja Costarricense de Seguro Social (HSJD-CCSS), San José, CRI; 3 Research Center in Hematology and Related Disorders, Costa Rica University, San José, CRI

**Keywords:** adult cardiac surgery, latin american healthcare, low- and middle-income country, minimally invasive cardiac surgery, perioperative mortality

## Abstract

Objective: To characterize the clinical and sociodemographic variables of patients undergoing cardiac surgery at Hospital San Juan de Dios-Caja Costarricense de Seguro Social (HSJD-CCSS) between 2016 and 2020, and to identify predictors of in-hospital mortality.

Materials and methods: A retrospective, observational study was conducted, including all patients ≥13 years who underwent cardiac surgery during the study period (n = 560). Sociodemographic variables, comorbidities, cardiovascular risk factors, European System for Cardiac Operative Risk Evaluation (EuroSCORE), New York Heart Association (NYHA) class, brain natriuretic peptide (BNP), type and urgency of surgery, use of cardiopulmonary bypass (CPB), and outcomes (complications, mortality, hospital and ICU stay) were collected. Descriptive statistics, association tests, and multivariable logistic regression models were applied; in subgroups with a low number of cases (endocarditis, aortic pathology), Firth regression was used.

Results: The median age was 59 years, with a predominance of males (57.9%). The main surgical indication was valvular disease (55.2%), followed by coronary artery disease (25%). Although overall mortality decreased from 14.9% (2010-2015) to 11.6% in the current period, the change was not statistically significant. In the global model, independent predictors of mortality were elevated BNP levels, emergency surgery, and prolonged ICU stay. In subgroup analyses, mortality was associated with BNP and NYHA in valvular disease; age, BMI, and surgical urgency in coronary artery disease; BNP, BMI, CPB, and minimally invasive surgery (MIS) in endocarditis; and CPB time in aortic pathology.

Conclusions: In-hospital mortality in cardiac surgery at HSJD decreased compared to the previous five-year period. The identification of pathology-specific predictors supports the need for individualized approaches and optimization of critical resources to improve surgical outcomes in Costa Rica.

## Introduction

Cardiovascular diseases are the leading cause of mortality worldwide [1], exerting a significant strain on health systems, particularly in developing nations [2,3]. In Costa Rica, ischemic heart disease remains one of the main causes of premature mortality [4,5], highlighting the critical need for specialized cardiac surgery services to enable timely and effective management of these conditions.

Cardiac surgery has undergone significant advancements in recent decades, including improvements in surgical techniques, the introduction of novel devices, and enhancements to perioperative care protocols [6]. These developments have collectively contributed to reductions in procedure-related mortality and complications, improved patient recovery, and more efficient consumption of hospital resources [7]. Nevertheless, despite these global improvements, data on patients undergoing cardiac surgery in Costa Rica remain scarce, limiting the ability to design and implement context-specific strategies for quality improvement [8,9].

The Hospital San Juan de Dios (HSJD) is one of the national referral centers for cardiac surgery within the Costa Rican Social Health Security Fund (Caja Costarricense de Seguro Social (CCSS)). Given its surgical volume and the diversity of cases, HSJD serves as a critical source of data for evaluating trends and outcomes in cardiac surgery at the national level [8-10]. Despite its importance, until recent years, there was a notable absence of studies systematically documenting the clinical and sociodemographic characteristics of patients undergoing cardiac surgery at this hospital [11].

In an effort to fill this gap, an initial characterization study of patients undergoing cardiac surgery at HSJD between 2010 and 2015 was conducted. This study represented a crucial starting point for the analysis of surgical outcomes, the identification of risk factors associated with mortality, and the evaluation of trends in surgical practice within the hospital. The findings obtained allowed for the establishment of reference parameters in terms of complication rates, mortality predictors, and patterns of postoperative morbidity, and to compare them with those described in similar settings [8,12].

The previous study at HSJD (2010-2015) found that most cardiac surgery patients were male, with an average age of 57.1 years. Valve replacement was the most common procedure, followed by coronary revascularization. The overall mortality rate was 14.9%, particularly higher in emergency cases. Key mortality predictors included a high European System for Cardiac Operative Risk Evaluation (EuroSCORE), elevated brain natriuretic peptide (BNP) levels, and advanced New York Heart Association (NYHA) class [8]. These insights permitted an update in institutional guidelines to enhance risk assessment and perioperative management. Building on previous research, the present study analyzes data from patients who underwent cardiac surgery at HSJD between 2016 and 2020. This analysis aims to enhance understanding of the clinical management and demographic characteristics of the patient population during this period, while also identifying new opportunities for improving patient care.

## Materials and methods

This was a retrospective observational cohort study that included all patients aged 13 years and older who underwent cardiac surgery at HSJD-CCSS, extending from the date of surgical intervention until hospital discharge or in-hospital death, between January 1, 2016, and December 31, 2020. Exclusion criteria included patients younger than 13 years, those evaluated by the cardiac surgery department but not operated on, and individuals who underwent surgery outside the study period. A total of 560 patients were included. A descriptive, retrospective analysis was conducted to evaluate their clinical and sociodemographic characteristics. Patients were categorized based on the type of surgical procedure performed, including valvular surgery, coronary artery bypass grafting (CABG), combined coronary-valvular procedures, ascending aorta and aortic arch surgery, and adult congenital heart disease, among others. Data collected for each patient included age, sex, place of residence, occupation, comorbidities, diagnosis, body mass index (BMI), cardiovascular risk factors, left ventricular ejection fraction (LVEF), length of hospital stay, intensive care unit (ICU) days, postoperative days, surgical mortality, EuroSCORE, NYHA functional class, preoperative BNP levels, type of surgery, specific procedure performed, and use of cardiopulmonary bypass (CPB).

All data was obtained from the department’s internal database and patient medical records. The study protocol was approved by the hospital's scientific ethics committee (CEC-HSJD-05-2022).

Statistical analysis

Statistical analyses were performed using IBM SPSS Statistics for Windows, Version 24 (Released 2016; IBM Corp., Armonk, New York, United States), SigmaStat for Windows Version 3.5 (Systat Software, Inc., San José, CA, USA), and GraphPad Prism version 8.0.1 (GraphPad Software, San Diego, CA, USA). Both descriptive and inferential statistics were used to characterize the study population.

Continuous variables were expressed as mean ± standard deviation (SD) for normally distributed data or as median and interquartile range (IQR) for non-normally distributed data. T-tests were used for comparisons between normally distributed continuous variables. Categorical variables were presented as frequencies and percentages, and significant associations between them were determined using chi-square tests or Fisher’s exact tests, as appropriate. In all cases, p-values were two-tailed, and values < 0.05 were considered significant.

In the overall population, independent predictors of mortality were identified using multivariable logistic regression analyses with backward selection. Results are reported as odds ratios (ORs) with 95% confidence intervals (CIs), and only complete datasets were examined.

Additionally, multivariable logistic regression with backward elimination was used to assess the association between clinical and surgical variables and in-hospital mortality for valvular disease and coronary artery disease. To maintain an adequate amount of data in these analyses, missing values were imputed. Missing continuous variables were replaced with the mean of the observed values, while missing categorical variables were imputed using the most frequent value. Since the events-per-variable criterion was not met in the analyses of bacterial endocarditis and aortic pathology, and to prevent overfitting, coefficient instability, and low statistical power, Firth logistic regressions were performed to identify predictor variables and estimate the probability of death in these cases. No missing data were present in the above-mentioned subsets.

## Results

General information

Between 2016 and 2020, a total of 560 patients underwent cardiac surgery, averaging 112 procedures per year, with a peak of 150 surgeries in 2016. The study population consisted of 324 (57.9%) males and 236 (42.1%) females, yielding a male-to-female ratio of approximately 1.4:1. The median age was 59.0 years (IQR: 50.0-67.0).

There was a statistically significant difference in age distribution between male and female patients. Men had a higher median age of 60.5 years (IQR: 53.0-68.0) compared to women, whose median age was 57.0 years (IQR: 46.0-66.0). Given that the data did not follow a normal distribution, the Mann-Whitney U test was used to compare the groups. The result was statistically significant (U = 43,425; T = 60,445; p = 0.004), indicating that the difference in age between sexes is unlikely to be due to chance and may reflect meaningful clinical or demographic variation in the population.

Most patients resided in San José, 413 (73.8%), followed by Alajuela, 65 (11.6%), and Puntarenas, 52 (9.3%). Regarding employment status, 228 (40.7%) were actively working, 123 (22.0%) were retired, and 140 (25.0%) of women were engaged in domestic work. Table 1 summarizes the general characteristics of the study population.

**Table 1:** General characteristics of the study population ICU: intensive care unit; N/A: not available; IQR: interquartile range

General Characteristics	Count (560 patients)
Sex	-
Female	236 (42.1%)
Male	324 (57.9%)
Age (Median IQR))	-
Male	60.5 (IQR: 53.00 - 68.00)
Female	57.0 (IQR: 46.0 - 66.0)
Total	59.0 (IQR: 50.0 - 67.0)
Employment Status	-
Active worker	228 (40.7%)
Domestic work	140 (25.0%)
Retired	123 (22.0%)
Unemployed	48 (8.6%)
Student	11 (2.1%)
N/A	10 (1.8%)

The mean BMI of the study population was 27.2 kg/m². Based on standard BMI classification, 15 (2.7%) of patients were underweight, 172 (30.7%) had normal weight, 193 (34.3%) were overweight, and 134 (24.0%) had some degree of obesity. Regarding preoperative LVEF, 257 (45.9%) of patients had normal ventricular function, while the remainder showed varying degrees of dysfunction. According to the NYHA functional classification, 157 (28.0%) of patients were in Class I, 213 (38.0%) in Class II, 96 (17.1%) in Class III, and 41 (7.3%) in Class IV. Additionally, 171 (30.5%) of patients had a preoperative BNP level classified within the intermediate range (100-500 pg/mL). The most common cardiovascular risk factors were hypertension, affecting 315 individuals (56.3%), and diabetes mellitus, affecting 240 individuals (44.2%). Table 2 summarizes the general preoperative clinical characteristics of the study population.

**Table 2:** Preoperative characteristics BMI: body mass index; LVEF: left ventricular ejection fraction; NYHA: New York Heart Association; BNP: brain natriuretic peptide; HTN: hypertension

Preoperative Characteristics	Count (560 patients)
BMI (kg/m²)	-
Underweight (<18.5)	15 (2.7%)
Normal weight (18.5-24.9)	172 (30.7%)
Overweight (25-29.9)	193 (34.3%)
Obesity I (30-35)	100 (17.3%)
Obesity II (35-40)	21 (3.8%)
Obesity III (>40)	13 (2.3%)
Not reported	46 (8.2%)
LVEF (%)	-
<30 (Severe dysfunction)	10 (1.8%)
30-40 (Moderate dysfunction)	42 (7.5%)
41-55 (Mild dysfunction)	186 (33.2%)
>55 (Normal function)	257 (45.9%)
Not reported	65 (11.6%)
NYHA Functional Class	-
Stage I	157 (28.0%)
Stage II	213 (38.0%)
Stage III	96 (17.1%)
Stage IV	41 (7.3%)
Not reported	53 (9.5%)
Preoperative BNP (pg/dL)	-
<100 (Normal range)	252 (45.0%)
100-500 (Intermediate range)	171 (30.5%)
>500 (High risk)	55 (9.8%)
Not reported	82 (14.6%)
Cardiovascular Risk Factors	-
Hypertension	315 (56.3%)
Diabetes mellitus	240 (44.2%)
Dyslipidemia	220 (39.3%)
Sedentary lifestyle	124 (22.1%)
Smoking	50 (8.9%)

**Figure 1:**
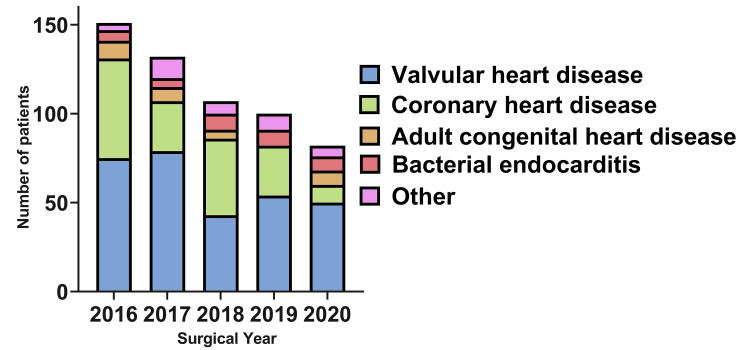
Annual distribution of admission diagnoses among cardiac surgery patients (2016-2020)

**Figure 2:**
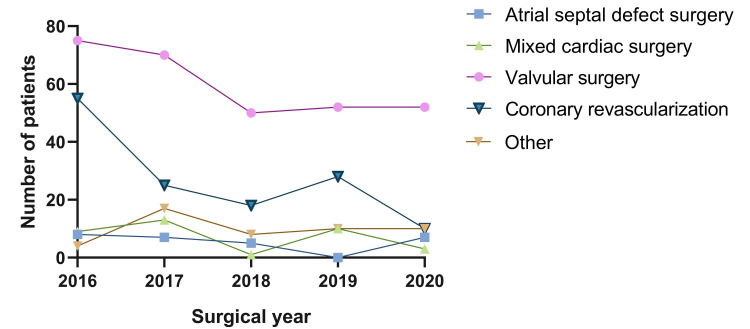
Yearly trends in types of cardiac surgeries performed (2016-2020)

The main admission diagnoses reported throughout the study period were valvular heart disease, 309 (55.2%); coronary artery disease, 140 (25.0%); bacterial endocarditis, 37 (6.6%); adult congenital heart disease, 31 (5.5%); cardiac myxoma, 13 (2.3%); and aortic pathology, 12 (2.1%). Figure 1 illustrates the annual distribution of these diagnoses, stratified by type, while Figure 2 displays trends over time based on the type of cardiac surgery performed.

In terms of surgical priority, 483 (86.3%) of the study population underwent elective procedures, while 74 (13.2%) required emergency surgery. The median hospital stay was 12.0 days (IQR: 8.0-20.0), with a median postoperative stay of 6.0 days (IQR: 5.0-10.0). The median duration of ICU stay was 2.0 days (IQR: 1.0-4.0).

The majority of cardiac surgeries performed, 309 (55.2%), involved valvular surgery, and CPB was utilized in 426 (76.1%) of all surgical procedures, with a median duration of 112 minutes (IQR 90.00-143.25).

Overall mortality

The overall mortality rate in our study population was 65 (11.6%). To explore potential predictors of postoperative mortality, a multivariate logistic regression analysis was performed using only complete datasets. Candidate variables were selected based on prior literature, and predictors were considered statistically significant if they reached a p-value < 0.05 in any step of the modeling process.

The adjusted R² values across models ranged from 0.073 to 0.079, indicating that the final model explained approximately 7.5% of the variability in postoperative mortality. Although the explanatory power was modest, the variables identified may still have clinical relevance for preoperative risk stratification.

In the initial model (Model 1), longer ICU stay (β = 0.014, p = 0.002) and emergency surgery (β = 0.143, p = 0.008) were significantly associated with mortality. Preoperative BNP levels showed a trend toward significance (p = 0.072), whereas EuroSCORE, age, sex, and NYHA functional class were not significant predictors.

In the final model (Model 6), which included only statistically significant variables, three independent predictors of mortality were identified: elevated preoperative BNP (β = 0.058, p = 0.006), longer ICU stay (β = 0.014, p = 0.003), and emergency surgical indication (β = 0.156, p = 0.002) (Table 3). Model calibration was assessed using the Hosmer-Lemeshow goodness-of-fit test. Non-significant test results in the early steps (e.g., Step 1: χ² = 5.669, df = 8, P = 0.684; Step 4: χ² = 7.910, df = 8, P = 0.442) suggested adequate fit. However, Steps 5 and 6 showed a significant lack of fit (χ² = 21.379 and 19.619, respectively; both P < 0.05), indicating potential issues with calibration in the final model.

**Table 3:** Factors associated with mortality identified by multivariate logistic regression * = shows statistical significance; + = marginal significance; B: unstandardized coefficient; β: standardized coefficient; T: T-statistic; BNP: brain natriuretic peptide; NYHA: New York Heart Association; ICU: intensive care unit; EuroSCORE: European System for Cardiac Operative Risk Evaluation

Model	Variable	B	Standard Error	Beta	t	p-value
1	(Constant)	-0.200	0.094	-	-2.140	0.033
	Preoperative BNP	0.043	0.024	0.098	1.801	0.072+
	EuroSCORE	-0.001	0.020	-0.003	-0.070	0.944
	Gender	-0.006	0.030	-0.010	-0.213	0.831
	Age	0.001	0.001	0.023	0.482	0.630
	NYHA	0.023	0.016	0.067	1.416	0.157
	Days in ICU	0.014	0.005	0.145	3.044	0.002*
	Emergency or elective	0.143	0.053	0.137	2.684	0.008*
6	(Constant)	-0.144	0.055	-	-2.602	0.010*
	Preoperative BNP	0.058	0.021	0.132	2.738	0.006*
	Days in ICU	0.014	0.005	0.142	2.999	0.003*
	Emergency or elective	0.156	0.051	0.149	3.062	0.002*

**Figure 3:**
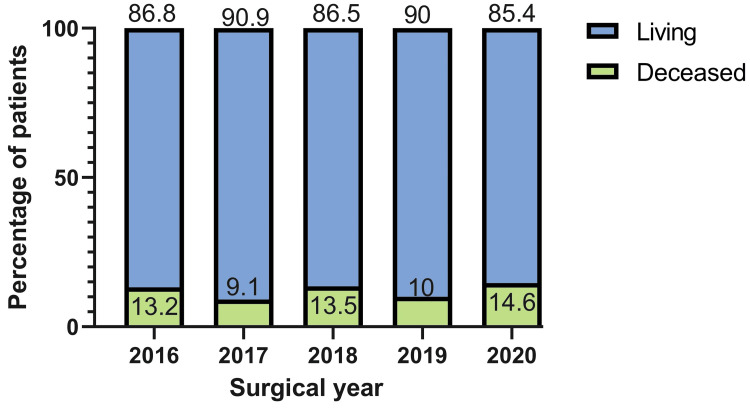
Annual distribution of living and deceased patients by surgical year (2016-2020)

**Figure 4:**
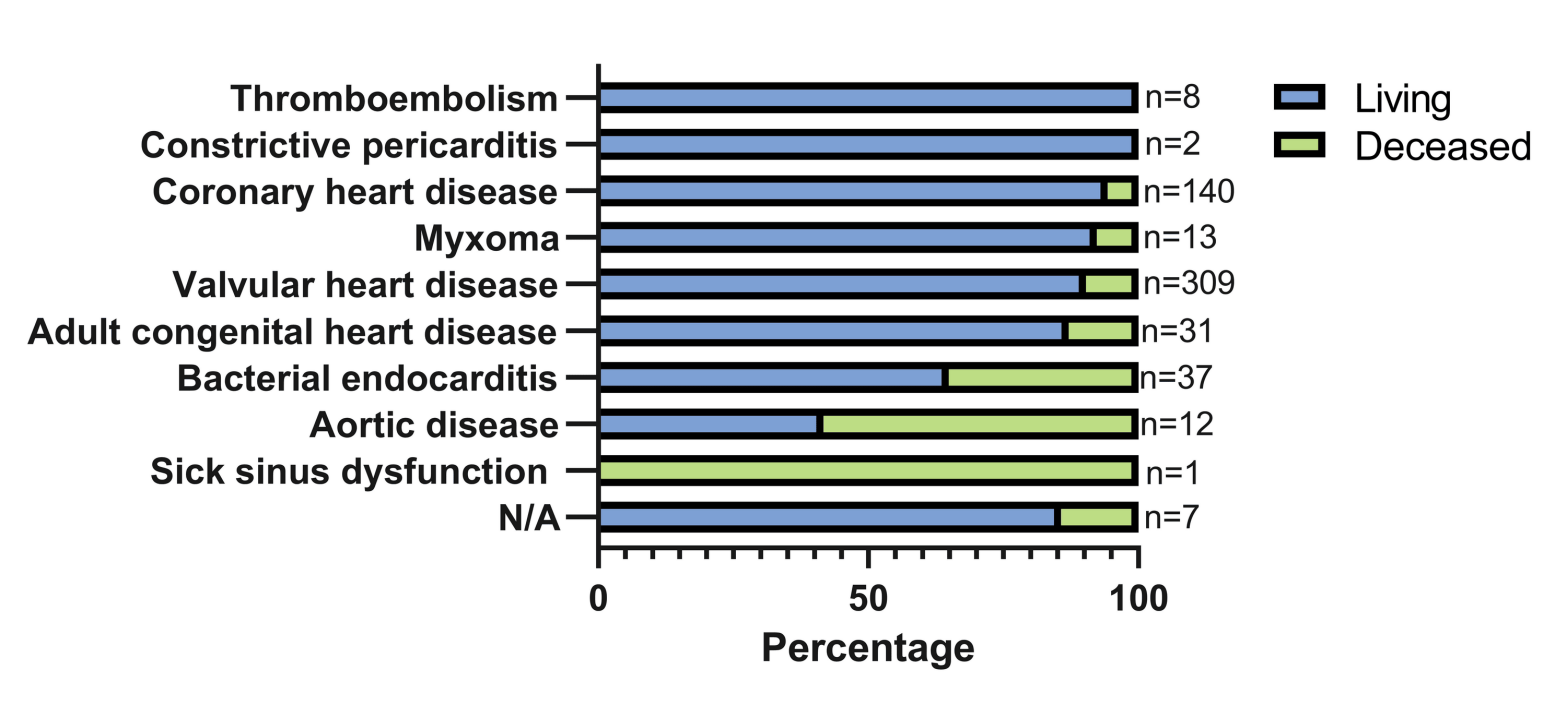
Distribution of patient outcomes (surviving vs. deceased) according to preoperative diagnosis N/A: not available

Although the total number of surgical cases steadily declined from 151 in 2016 to 82 in 2020, the absolute number of deceased patients remained relatively stable, ranging from 10 to 20 deaths per year. This trend resulted in a progressive increase in the proportion of postoperative mortality across the years. In 2016, deceased patients represented approximately 20 (13.2%) of the surgical cohort, while by 2020, this proportion had risen to 12 (14.6%) (Figure 3). On the other hand, Figure 4 displays the proportion of surviving and deceased patients by preoperative diagnosis. Certain conditions, such as bacterial endocarditis, aortic disease, and sick sinus dysfunction, were associated with higher mortality rates compared to others, like valvular or congenital heart disease.

Valvular disease mortality

To identify independent predictors of mortality in patients undergoing surgery for valvular heart disease, a multivariate logistic regression analysis was performed. The initial model (Model 1) included clinically relevant preoperative variables based on existing literature: preoperative BNP, EuroSCORE, NYHA functional class, use of CPB, length of stay in the ICU, LVEF, and surgical urgency (elective vs. emergency).

A stepwise selection procedure was employed to refine the model by excluding variables that did not contribute significantly. In the final model (Model 7), only preoperative BNP (β = 0.163, p = 0.017) and NYHA functional class (β = 0.132, p = 0.053) remained independently associated with mortality. These findings suggest that elevated preoperative BNP levels and a more advanced functional class may be indicative of increased perioperative risk in patients with valvular heart disease (Table 4). The adjusted R² of the model improved modestly from 0.025 in the initial model to 0.034 in the final model. Despite the modest explanatory power, the inclusion of BNP and NYHA class provides potentially valuable parameters for preoperative risk stratification. Model calibration was assessed using the Hosmer-Lemeshow goodness-of-fit test across multiple steps of the regression process. In all cases, the test was non-significant (e.g., Step 1: χ² = 6.334, df = 8, p = 0.610; Step 6: χ² = 3.914, df = 5, p = 0.562), indicating a satisfactory fit between the predicted probabilities and observed outcomes and supporting the overall validity of the model.

**Table 4:** Independent predictors of mortality identified by multivariate logistic regression in patients with valvular heart disease * = shows statistical significance; + = marginal significance; B: unstandardized coefficient; β: standardized coefficient; T: T-statistic; BNP: brain natriuretic peptide; NYHA: New York Heart Association; CPB: cardiopulmonary bypass; ICU: intensive care unit; LVEF: left ventricular ejection fraction; EuroSCORE: European System for Cardiac Operative Risk Evaluation

Model	Variable	B	Standard Error	Beta	t	p-value
1	(Constant)	-0.121	0.253	-	-0.477	0.634
	Preoperative BNP	0.070	0.035	0.165	1.983	0.049*
	EuroSCORE	0.025	0.029	0.061	0.881	0.380
	NYHA	0.044	0.022	0.137	1.967	0.051+
	CPB	0.000	0.000	0.060	0.867	0.387
	ICU days	0.010	0.008	0.093	1.346	0.180
	LVEF	0.000	0.002	0.009	0.124	0.902
	Elective or emergent	-0.012	0.200	-0.005	-0.059	0.953
7	(Constant)	-0.036	0.052	-	-0.684	0.495
	Preoperative BNP	0.069	0.029	0.163	2.414	0.017*
	NYHA	0.042	0.022	0.132	1.949	0.053*

Coronary artery disease mortality

A multivariate logistic regression analysis with backward stepwise elimination was conducted to determine predictors of post-surgical mortality among patients with coronary artery disease. The initial model (Model 1) incorporated a range of clinically relevant preoperative variables, including BNP, EuroSCORE, sex, age, BMI, ICU length of stay, and surgical urgency (emergency vs. elective), as guided by prior literature [13,14].

In this first iteration, BMI (β = 0.225, p = 0.015) and age (β = 0.189, p = 0.049) were significantly associated with mortality, while BNP, EuroSCORE, and ICU days showed no statistical relevance. The model’s fit was moderate, with an R² of 0.168, indicating that approximately 16.8% of the variance in mortality was explained by the variables included. Additionally, a significant F-change (p = 0.012) supported the relevance of the initial set of variables. As the model was refined through successive steps (Models 2 to 6), non-contributory variables were systematically excluded. In the final model (Model 6), three independent predictors remained: age (β = 0.200, p = 0.034), BMI (β = 0.198, p = 0.029), and emergency surgical indication (β = 0.214, p = 0.022). These variables consistently demonstrated the strongest association with postoperative mortality in this patient group. While the final model’s R² was slightly lower (0.138) and the adjusted R² remained modest (0.114), the retained variables reflected a more parsimonious and clinically interpretable model. The lack of a statistically significant F-change in the final step (p = 0.163) indicated that the reduction in variables did not meaningfully affect the model’s overall performance (Table 5). Calibration of the logistic model was assessed using the Hosmer-Lemeshow test. All iterations of the model yielded non-significant results (e.g., Step 1: χ² = 5.875, df = 8, p = 0.661; Step 5: χ² = 4.396, df = 8, p = 0.820), confirming good agreement between predicted and observed outcomes and supporting the adequacy of the model’s fit to the data.

**Table 5:** Results of multivariate logistic regression analysis for mortality in coronary artery disease * = shows statistical significance; + = marginal significance; B: unstandardized coefficient; β: standardized coefficient; T: T-statistic; BNP: brain natriuretic peptide; NYHA: New York Heart Association; ICU: intensive care unit; BMI: body mass index; EuroSCORE: European System for Cardiac Operative Risk Evaluation

Model	Variable	B	Standard Error	Beta	t	p-value
1	Constant	-0.643	0.176	-	-3.650	0.000
	Preoperative BNP	0.027	0.056	0.070	0.482	0.631
	EuroSCORE	-0.039	0.026	-0.134	-1.473	0.144
	Sex	0.055	0.052	0.096	1.045	0.298
	Age	0.005	0.002	0.189	1.995	0.049*
	ICU days	0.003	0.012	0.023	0.237	0.813
	BMI	0.058	0.024	0.225	2.462	0.015*
	Emergency or elective	0.179	0.091	0.192	1.959	0.053+
6	Constant	-0.624	0.172	-	-3.629	0.000
	Emergency or elective	0.199	0.086	0.214	2.316	0.022*
	BMI	0.051	0.023	0.198	2.214	0.029*
	Age	0.005	0.002	0.200	2.147	0.034*

Bacterial endocarditis mortality

To assess mortality predictors in patients with bacterial endocarditis who underwent surgical management, a stepwise multivariate Firth logistic regression was conducted using three sequential models. Predictor variables were selected based on previously established clinical relevance in the literature [15].

The first model (Model 1) incorporated seven covariates: preoperative BNP, age, BMI, ICU length of stay, CPB, EuroSCORE, and minimally invasive surgery (MIS). This model demonstrated strong explanatory capacity, with excellent calibration (deviance p = 1.000, Pearson p = 0.997). Within this model, BNP (p = 0.002), BMI (p = 0.037), and CPB (p = 0.015) emerged as significant predictors, while ICU length of stay approached statistical significance (p = 0.058).

As non-significant variables were progressively removed, the final model (Model 3) retained five covariates: BNP, BMI, CPB, ICU stay, and MIS. This iteration maintained perfect model fit (deviance p = 1.000, Pearson p = 0.981). Notably, BNP (β = 0.796, p < 0.001), CPB (β = 0.512, p = 0.012), BMI (β = -0.421, p = 0.023), and MIS (β = -0.488, p = 0.042) remained statistically significant, while ICU days demonstrated marginal significance (p = 0.053). Age and EuroSCORE II were excluded due to a lack of contribution to the model (Table 6). Model calibration, evaluated using deviance and Pearson χ² statistics, showed a perfect fit across all steps, with p-values ≥ 0.981 in every iteration. This reflects excellent agreement between observed and predicted mortality, supporting the robustness and reliability of the final regression model.

**Table 6:** Mortality predictors in bacterial endocarditis (results from stepwise Firth logistic regression) * = shows statistical significance; + = marginal significance; BNP: brain natriuretic peptide; BMI: body mass index; CPB: cardiopulmonary bypass; ICU: intensive care unit; MIS: minimally invasive surgery; EuroSCORE: European System for Cardiac Operative Risk Evaluation

Model	Variable	B	Standard Error	β	χ²	p-value
1	(Constant)	5.124	5.012	-	0.597	0.440
	BNP	0.001	0.000	0.639	9.696	0.002*
	Age	-0.099	0.064	-0.174	1.908	0.167
	BMI	-1.688	0.937	-0.362	4.362	0.037*
	ICU days	0.118	0.066	0.374	3.597	0.058+
	EuroSCORE	-1.188	1.531	-	0.340	0.560
	CPB	0.031	0.016	0.460	5.953	0.015*
	MIS	-2.369	1.668	-	2.321	0.128
3	(Constant)	-1.786	1.937	-	0.608	0.435
	BNP	0.001	0.000	0.796	10.891	<0.001*
	BMI	-1.261	0.647	-0.421	5.166	0.023*
	ICU days	0.088	0.050	0.316	3.735	0.053+
	CPB	0.025	0.011	0.512	6.343	0.012*
	MIS	-2.873	1.663	-0.488	4.124	0.042*

Mortality in aortic pathology

To evaluate mortality predictors of in-hospital patients with aortic pathology, a stepwise Firth’s biased-reduced logistic regression analysis was performed. The choice of Firth’s penalized likelihood approach was based on the very limited sample size (n = 12), which increases the risk of small-sample bias and convergence issues in conventional maximum likelihood logistic regression. The first model (Model 1) incorporated eight clinically relevant covariates identified from the literature: CPB, EuroSCORE, emergency versus elective surgery (ERoEL), primary diagnosis (ascending aortic aneurysm, Crawford type 1 thoracoabdominal aneurysm, and aortic dissection type A), MIS, BMI, ICU length of stay, and sex [16,17]. Although no predictors reached conventional statistical significance in this full model, through a sequential stepwise process, non-contributory variables were removed to improve model parsimony and reduce the risk of overfitting. The final retained model (Model 6) included only three variables, CPB, EuroSCORE, and ERoEL, which collectively demonstrated improved overall model fit (χ² = 13.92, p = 0.003). Importantly, model calibration remained excellent, as shown by the goodness-of-fit statistics: the deviance test yielded a p-value of 0.000, and the Pearson test a p-value of 0.773, both far above the conventional significance threshold. These values indicate perfect agreement between observed and predicted outcomes, suggesting that the model reliably estimates mortality risk within this dataset despite its small sample size.

Within this final model, CPB emerged as a statistically significant predictor of mortality (p = 0.039), suggesting that prolonged bypass times may adversely impact postoperative survival in this high-risk, small-sample population. EuroSCORE, a widely used surgical risk stratification tool, displayed marginal significance (p = 0.054), consistent with its established role as a composite indicator of perioperative mortality risk. ERoEL also approached significance (p = 0.085), with emergency procedures tending toward higher mortality compared to elective cases (Table 7). The decision to halt stepwise selection at Model 6 was deliberate, as further elimination of variables resulted in the loss of statistical significance for the remaining predictors. This pattern suggests that there may be important interactions or synergistic effects among CPB, EuroSCORE, and ERoEL, whereby their combined influence better explains mortality risk than any single factor in isolation. Given the very limited sample size, these findings should be interpreted with caution, but they provide clinically relevant insights that align with existing literature on the adverse prognostic impact of prolonged CPB duration and urgent surgical indications. These results highlight the importance of optimizing surgical planning, minimizing bypass time, and prioritizing elective intervention where possible to improve outcomes in this vulnerable subgroup.

**Table 7:** Mortality predictors in aortic pathology (results from stepwise Firth logistic regression) *: shows statistical significance; + = marginal significance; BNP: brain natriuretic peptide; BMI: body mass index; CPB: cardiopulmonary bypass; ICU: intensive care unit; MIS: minimally invasive surgery; ERoEL: emergency versus elective surgery; EuroSCORE: European System for Cardiac Operative Risk Evaluation

Model	Variable	B	Standard Error	β (Beta)	χ² / t	p-value
1	(Intercept)	4.749	5.800	-	0.677	0.411
	CPB	0.032	0.021	-	2.988	0.084
	EuroSCORE	1.124	1.164	-	1.013	0.314
	ERoEL	-3.789	2.171	-	3.639	0.056
	Diagnoses	0.196	1.555	-	0.012	0.913
	MIS	0.978	1.453	-	0.419	0.517
	BMI	-0.941	0.987	-	0.953	0.329
	ICU days	-0.160	0.211	-	0.526	0.468
	Sex	-2.610	2.195	-	1.605	0.205
6	(Intercept)	0.562	2.339	-	0.049	0.825
	CPB	0.021	0.012	-	4.247	0.039*
	EuroSCORE	2.563	1.686	-	3.710	0.054+
	ERoEL	-3.599	2.374	-	2.961	0.085

## Discussion

This study offers an updated and comprehensive characterization of patients undergoing cardiac surgery at HSJD-CCSS in Costa Rica between 2016 and 2020. As one of the country's principal public referral centers for cardiovascular surgery, HSJD-CCSS plays a central role in national surgical outcomes, and the present analysis serves both as a reflection of clinical trends and as a benchmark for evaluating institutional progress over time.

The demographic profile of the study population remains consistent with previous national results and international literature. The predominance of male patients, with a mean age of approximately 57 years, aligns with global patterns reported in high-volume cardiovascular surgery centers [15,18]. Most interventions were elective, with valvular surgeries and CABG constituting the majority of procedures. These findings mirror the surgical distribution reported by Lotz-Esquivel et al. (2019) for the 2010-2015 period, suggesting that the case mix and demographic structure of cardiac surgery patients at HSJD have remained relatively stable over the past decade [8].

A key finding of the current analysis is the observed reduction in overall in-hospital mortality, which decreased from 14.9% in the 2010-2015 period to 11.6% between 2016 and 2020. While this decline may appear modest, it likely reflects incremental yet meaningful improvements in perioperative care, surgical technique, and patient selection [8,19]. It is noteworthy, however, that mortality did not decline linearly across the five-year span; in fact, the later years of the period saw a slight increase in case fatality, suggesting potential shifts in case complexity, patient comorbidities, or healthcare system strain that merit further exploration.

The identification of independent predictors of mortality is one of the central contributions of this study. In multivariable logistic regression models, emergency surgical indication, elevated preoperative BNP levels, and prolonged ICU stay emerged as the most significant predictors of postoperative death. These findings are consistent with existing evidence in the literature, including the earlier HSJD study by Lotz-Esquivel et al., which also reported elevated BNP and emergency surgery as strong prognostic markers [8]. However, there are notable distinctions between the two studies: while the 2010-2015 analysis highlighted EuroSCORE and NYHA functional class as primary predictors, these variables did not retain statistical significance in our final models. Instead, our results suggest a gradual shift away from static preoperative risk scores toward more dynamic clinical and perioperative indicators, such as hemodynamic parameters and critical care burden [20,21].

Unlike its predecessor, the present study incorporated subgroup-specific regression models stratified by preoperative diagnosis, namely, coronary artery disease, valvular disease, endocarditis, and aortic pathology. These subgroup analyses revealed diagnosis-specific predictors: age, BMI, and surgical urgency were most relevant in coronary artery disease; BNP, CPB, BMI, and minimally invasive approach were significant in endocarditis; and in aortic pathology, CPB, EuroSCORE, and urgency dominated the predictive landscape. These distinctions support the growing consensus that perioperative risk is highly context-dependent and that generic models may fail to capture the heterogeneity of cardiac surgery patients [22,23].

It is important, however, to consider the effect of sample size on these subgroup analyses. In the case of endocarditis, the subgroup comprised 37 patients, substantially larger than the aortic pathology subgroup, yet still relatively small for multivariable modeling. While BNP, CPB, BMI, and MIS emerged as significant predictors, the modest sample size limits statistical power and raises the possibility that other clinically meaningful predictors were excluded. These findings are therefore best interpreted as exploratory and hypothesis-generating, requiring confirmation in larger cohorts.

For aortic pathology, the challenge was even greater: this subgroup included only 12 patients, reflecting the rarity and clinical severity of conditions such as thoracoabdominal aortic aneurysm and acute aortic dissection. Although CPB was significant and EuroSCORE and urgency approached significance, the very limited number of cases inevitably constrains the robustness of these associations. In this setting, the model serves primarily to highlight plausible prognostic signals, such as the detrimental effect of prolonged bypass times, rather than to establish definitive predictors. The small sample size increases susceptibility to model instability, overfitting, and spurious associations, underscoring the need for multicenter validation before these findings can inform practice, so these results are only exploratory.

Further comparison with the findings of Matarrita Quesada et al. (2025), also conducted at HSJD, reinforces the validity of our conclusions. That study identified ICU readmission (OR = 12.7), reintervention (OR = 7.1), and prolonged CPB time (OR = 4.7) as the strongest predictors of mortality [9]. Although our analysis did not include ICU readmission or reintervention as variables, it did confirm the predictive importance of CPB in subgroup analyses and the critical prognostic value of prolonged ICU stays. Importantly, our study also identified preoperative BNP as a significant mortality predictor, a variable not assessed in the 2024 study. These differences may be attributable to variation in study design, available variables, and surgical protocols. Nonetheless, both studies converge on the importance of perioperative complexity and resource use as key determinants of outcome, particularly in publicly funded, resource-limited settings such as the Costa Rican Social Health Security Fund.

From an international perspective, the study by Pelliccia et al. (2019) on patients with myocardial infarction and non-obstructive coronary arteries (MINOCA) provides a compelling point of comparison, despite differences in patient population and surgical context. Their meta-analysis identified reduced LVEF, beta-blocker use, and ST-segment depression as key predictors of long-term mortality [24]. The fact that reduced ejection fraction and elevated BNP levels, both markers of cardiac dysfunction, also predicted mortality in our cohort supports the broader applicability of these indicators across various forms of cardiovascular disease. Moreover, the increased mortality associated with emergency presentations in our study mirrors the negative prognostic weight of acute clinical status observed in the MINOCA population. These parallels reinforce the external validity of our findings and highlight the value of integrating acute physiological indicators into cardiac surgical risk models.

On a broader systemic level, our findings resonate with the global analysis presented by Megantara et al. (2025), who investigated the influence of health system infrastructure on cardiovascular outcomes across 183 countries [25]. Their results demonstrated that the absence of universal health coverage, national cardiovascular strategies, and primary care-based risk stratification programs were all associated with elevated mortality. While our study operates at the clinical rather than macroeconomic level, the correlation between emergency procedures, late-stage presentations, and poor outcomes in our population may reflect similar gaps in preventive care and early detection. In this light, our findings suggest that institutional improvements in preoperative triage, patient referral, and perioperative planning could yield significant benefits, not only by improving surgical outcomes but also by mitigating systemic inefficiencies that drive late interventions.

Despite the clinical relevance of our findings, the overall explanatory power of the statistical models remains modest. The adjusted R² values of the multivariable regressions ranged from 0.07 to 0.52, depending on the subgroup. While this variability reflects the multifactorial nature of postoperative mortality, it also underscores the limitations of current data sources [21,26]. Factors such as intraoperative complications, detailed surgical technique, surgeon experience, and postoperative care quality, none of which were included in our dataset, are likely to account for additional variance. Moreover, non-clinical variables such as socioeconomic status, health literacy, and social support may play a nontrivial role in postoperative recovery and mortality. These elements are particularly relevant in the Costa Rican public health context, where health equity remains a central institutional objective but is not always systematically measured in hospital-based research [25].

The study is strengthened by several key features, including its use of a comprehensive and standardized clinical database, large sample size overall, and methodologically rigorous statistical approach. The incorporation of subgroup-specific models, evaluation of model calibration using the Hosmer-Lemeshow test, and the use of backward stepwise variable selection represent methodological advancements compared to earlier local studies [8,26]. These techniques allowed for a more granular understanding of risk patterns and enhanced the clinical relevance of the results.

Nonetheless, the study is not without limitations. Its retrospective design introduces inherent risks of selection bias and information bias, particularly given the exclusion of patients operated on outside the defined timeframe or at other institutions. Additionally, missing data for certain variables, especially laboratory and echocardiographic parameters, may have introduced residual confounding. Future studies would benefit from prospective data collection, inclusion of intraoperative metrics, and linkage with national registries to enable long-term follow-up and capture of post-discharge events [3,26,27]. Most importantly, subgroup analyses with relatively small samples, such as endocarditis (n = 37) and especially aortic pathology (n = 12), should be interpreted with caution. Their main value lies in identifying potential predictors consistent with biological plausibility and prior evidence, but they remain hypothesis-generating and require confirmation in larger, multicenter cohorts.

Taken together, our findings offer a contemporary snapshot of cardiac surgery outcomes at HSJD-CCSS, one of Costa Rica’s leading cardiovascular centers. The decrease in in-hospital mortality, emergence of new clinical predictors, and refined modeling approaches collectively reflect an evolution in surgical practice and analytic sophistication. Importantly, the study identifies actionable targets for institutional improvement, namely, early risk detection (e.g., through BNP), management of high-risk presentations, and optimization of ICU resources. It also highlights the need for broader system-level interventions, such as strengthening referral pathways and investing in perioperative infrastructure, to reduce preventable mortality.

This study not only characterizes the current state of cardiac surgery at a major public hospital but also lays the groundwork for future institutional strategies. The integration of context-specific clinical indicators, such as BNP and ICU stay duration, into perioperative planning may enhance risk stratification and guide targeted interventions. At the same time, findings from smaller subgroups, particularly endocarditis (n = 37) and aortic pathology (n = 12), must be interpreted with caution, serving primarily as exploratory signals that require validation in larger studies. Continued benchmarking against both national and international data remains essential to drive quality improvement and ensure that Costa Rican cardiac surgical care continues to evolve in line with global standards.

## Conclusions

This study provides a detailed overview of patients who underwent cardiac surgery at HSJD between 2016 and 2020. Despite common comorbidities, the overall mortality rate improved compared to the previous period, suggesting advances in patient care and surgical management.

Key mortality predictors included high preoperative BNP, advanced NYHA class, longer ICU stays, and surgical urgency. Subgroup analyses highlighted distinct risk factors by pathology type, supporting the need for individualized treatment approaches. Although limited by its retrospective design, the study contributes valuable local data and underscores the importance of incorporating risk stratification and standardized protocols to improve surgical outcomes in Costa Rica.
